# *Underground allies*: Mycorrhizal flexibility facilitates orchid occurrence in post‐mining landscapes

**DOI:** 10.1111/plb.70269

**Published:** 2026-07-27

**Authors:** A. Ashokan, E. Sciaudone, A. Battaglini, P. Cortis, D. Cafasso, S. Cozzolino

**Affiliations:** ^1^ Department of Biology University of Naples Federico II, Complesso Universitario di Monte S. Angelo Naples Italy; ^2^ Independent Researcher Florence Italy; ^3^ Dipartimento di Scienze della Vita e dell'Ambiente Università degli Studi di Cagliari Cagliari Italy

**Keywords:** ecological restoration, *Epipactis helleborine*, fungal metabarcoding, genetic structure, habitat filtering, heavy metal contaminated soil, orchid mycorrhizal fungi, Orchidaceae, rhizosphere

## Abstract

- Orchids are generally regarded as ecological specialists because their dependence on obligate mycorrhizal symbioses and pollinators is thought to constrain them to relatively undisturbed habitats. However, the occurrence of some species in highly degraded environments challenges this assumption. *Epipactis helleborine*, a widespread Eurasian orchid, successfully colonizes heavy metal contaminated mining sites in Italy, where most vegetation fails to establish.
- We investigated whether the occurrence of *E. helleborine* in these extreme habitats is facilitated by shifts in fungal associations and examined the genetic structure underlying this ecological novelty. Fungal communities and orchid genomic structure were analysed across paired mining and woodland sites in Sardinia and Tuscany using fungal amplicon sequencing and plant ddRAD sequencing, respectively.
- While total fungal community composition showed only weak habitat‐related structuring, mycorrhizal communities exhibited moderate to strong differentiation between woodland and mining habitats. Woodland and mining populations shared several ectomycorrhizal associates, including *Balsamia,* while *Tuber* was markedly more abundant in woodlands and largely absent from mines, which instead harboured higher proportions of stress‐tolerant and opportunistic taxa. Despite these ecological differences, genomic analyses revealed little genetic differentiation between habitats, supporting repeated colonization and ongoing gene flow among populations.
- Our findings add to growing evidence that mycorrhizal flexibility facilitates the establishment and persistence of plants in novel or anthropogenically altered environments. The ability of *E. helleborine* to associate with diverse mycorrhizal partners may have contributed to its successful colonization of post‐mining habitats while maintaining genetic connectivity with nearby woodland populations.

## Graphical abstract

*Epipactis helleborine* associates with largely habitat‐specific fungal communities, with limited overlap between mining and woodland sites, yet maintains comparable genetic diversity across habitats. These results highlight the role of mycorrhizal flexibility in enabling habitat shifts and expanding ecological niches in human‐altered landscapes.

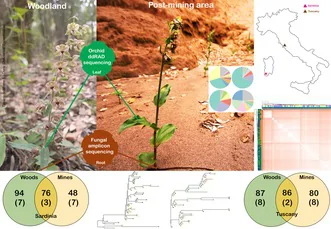

## INTRODUCTION

Ecological specialization often confines species to narrow realized niches, making them highly efficient in stable environments but seemingly ill‐suited to disturbed or extreme habitats (Futuyma & Moreno 1988; Carboni *et al*. 2016; Hirst *et al*. 2017; Gubry‐Rangin *et al*. 2024). Orchids (Orchidaceae Juss.), among the most diverse and evolutionarily successful families of flowering plants, exemplify this pattern and are widely recognized as ecological specialists (Cozzolino & Widmer 2005; McCormick & Jacquemyn 2014; Givnish *et al*. 2015; McCormick *et al*. 2018; Freudenstein 2025). Their survival hinges on a suite of intricate ecological dependencies, such as specialized pollinators (Cozzolino & Scopece 2008; Schiestl & Schlüter 2009) and obligate mycorrhizal associations that are required for seed germination and early development (McCormick & Jacquemyn 2014; Voyron *et al*. 2017; McCormick *et al*. 2018). Although many orchids have been associated with relatively undisturbed or stable habitats, this view is not universal. Numerous species occur in disturbed or semi‐natural environments such as grasslands, meadows, dune slacks, and road verges, and many are known to colonize anthropogenic habitats (Collins *et al*. 2005; Shefferson *et al*. 2008; Jacquemyn *et al*. 2016, 2020). Some orchid species have also been reported from industrial waste sites and abandoned mining areas characterized by extreme edaphic conditions, including heavy metal contamination, low nutrient availability, shallow substrates, and reduced vegetation cover (Shaw 1998; Collins *et al*. 2005; Shefferson *et al*. 2008; De Agostini *et al*. 2020; Maleva *et al*. 2022; Mirioba *et al*. 2024). These observations suggest that specific traits, particularly flexibility in mycorrhizal associations and high seed dispersion, may play an important role in enabling some orchids to establish in these non‐native habitats (Collins *et al*. 2005; Esfeld *et al*. 2008; Shefferson *et al*. 2008; Maleva *et al*. 2022; Yu *et al*. 2025).

**Fig. 1:**
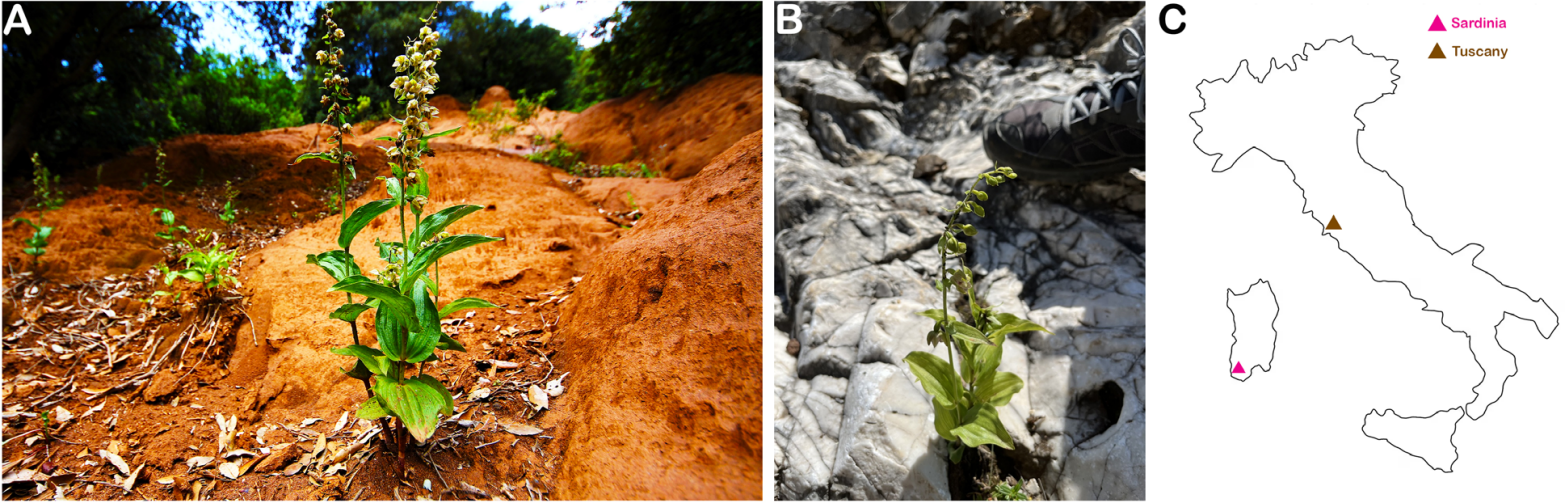
Study species and sampling locations. (A) *Epipactis helleborine* growing at a mining site in Sardinia, (B) *E. helleborine* at a mining site in Tuscany, and (C) Geographical map showing the locations of Sardinia and Tuscany in Italy.

The broad‐leaved helleborine, *Epipactis helleborine* (L.) Crantz (Orchidaceae: Epidendroideae: Neottieae) is a terrestrial orchid species that displays exceptional ecological breadth and geographic range (Fig. 1A,B; Ogura‐Tsujita & Yukawa 2008; Tranchida‐Lombardo *et al*. 2011; Jacquemyn *et al*. 2016; Djordjević *et al*. 2020; Xing *et al*. 2020; Valencia *et al*. 2021). It is one of the most widely distributed orchid species, which occurs from northwest Africa and Europe to East Asia (POWO 2026). *Epipactis helleborine* has also been introduced and naturalized in North America and occupies a wide spectrum of bioclimatic zones and soil types (Tešitelová *et al*. 2012; Rewicz *et al*. 2017; Račko *et al*. 2021). As a rhizomatous geophyte with marked trophic flexibility, *E. helleborine* spans a continuum from autotrophy to full mycoheterotrophy and is generally regarded as mixotrophic (Jacquemyn *et al*. 2016; Valencia *et al*. 2021). This nutritional plasticity is underpinned by associations with a broad array of mycorrhizal fungi, including diverse ectomycorrhizal species within Pezizales (Ogura‐Tsujita & Yukawa 2008; Jacquemyn *et al*. 2016). Together, trophic flexibility and broad symbiotic potential likely facilitate the species' ability to colonize both relatively stable natural habitats, such as woodland understories, and highly disturbed or extreme environments. Indeed, this orchid has been reported to occur both in urban environments (Rewicz *et al*. 2017) and in more extreme, polluted habitats, such as heavy metal contaminated mining sites (Fig. 1A,B; Collins *et al*. 2005; Shefferson *et al*. 2008).

There are currently no studies explicitly addressing colonization dynamics and gene flow between natural and highly disturbed anthropogenic habitats, such as post‐mining soils in orchids. However, previous work on *E. helleborine* in coastal dune systems has reported reduced genetic variation and low genetic differentiation between dune and forest populations (Jacquemyn *et al*. 2018, 2020; Evans & Jacquemyn 2020), suggesting that colonization of stressful environments may sometimes involve founder effects or demographic bottlenecks. However, coastal dunes and post‐mining habitats differ substantially in their ecological and historical context (Shefferson *et al*. 2008; De Agostini *et al*. 2020; Jacquemyn *et al*. 2020). Dune systems are relatively older, semi‐natural and more spatially continuous habitats, whereas abandoned mining sites represent recent anthropogenic environments characterized by strong edaphic discontinuities, heavy metal contamination, and highly localized disturbance regimes (Doing 1985; Shaw 1998; De Agostini *et al*. 2020). These differences in habitat history, spatial isolation, and environmental severity are likely to influence dispersal dynamics, population connectivity, and opportunities for local adaptation, potentially resulting in distinct genetic patterns across habitat types.

In Sardinia, a Mediterranean island characterized by long‐term geographic isolation, and Tuscany, a mainland region of central Italy (Fig. 1C), abandoned mining sites are characterized by toxic, nutrient‐poor, metal‐contaminated soils that are largely inhospitable to vascular plants (De Agostini *et al*. 2020; Bianchi *et al*. 2024). These disturbed habitats form sharp ecotones with adjacent remnant woodlands, generating strong environmental contrasts and potentially imposing distinct selective pressures on plant populations (De Agostini *et al*. 2020). Along these steep gradients, *E. helleborine* often persists as the only orchid species and, in some cases, the only vascular plant present (Fig. 1A,B), whereas nearby woodlands support diverse orchid assemblages including *Cephalanthera* Rich., *Ophrys* L., *Serapias* L., and additional *Epipactis* species. Although previous studies have investigated belowground fungal associations of orchids in comparable systems (Collins *et al*. 2005; Shefferson *et al*. 2008; De Agostini *et al*. 2020), it is well established that *E. helleborine* can associate with different mycorrhizal fungi across habitats, important uncertainties remain. In particular, it is unclear whether orchids in adjacent mine and woodland habitats share overlapping fungal partners or exhibit fine‐scale shifts in symbiotic associations in response to contrasting environmental conditions (Jacquemyn *et al*. 2016; Suetsugu *et al*. 2017). Moreover, it remains unknown whether colonization of extreme sites is driven by a limited number of genotypes or whether it involves multiple genetically diverse individuals.

To investigate the mechanisms underlying *E. helleborine*'s occurrence in challenging environments, we analysed the genetic structure of orchid individuals from both woodland and post‐mining sites in these two geographically distant locations, alongside their associated mycorrhizal and other fungal communities. Our goal was to explore potential links between symbiotic associations and genetic divergence across natural and disturbed habitats, and to determine whether only specific orchid genotypes can establish in these environments or if ongoing seed dispersal combined with habitat filtering enables persistence of certain genotypes. Evidence from other plant systems demonstrates that adaptation to extreme edaphic conditions often leads to pronounced genetic structuring within populations. For example, plant populations on serpentine soils show genetic differentiation shaped by isolation and drift, indicating multiple independent origins of serpentine tolerance (Westerbergh & Saura 1992; O'Dell & Rajakaruna 2011; Rajakaruna 2018; Konečná *et al*. 2020). Similarly, *Medicago lupulina* L. (Fabaceae) populations on serpentine substrates exhibit strong genetic divergence not explained by geographic distance, underscoring the role of edaphic selection in shaping diversity (Ahatović Hajro *et al*. 2024). Based on these observations, we hypothesize that the persistence of *E. helleborine* in mining areas is associated with both patterns of genetic variation and shifts in mycorrhizal associations that may facilitate establishment and survival in metal‐contaminated soils. Specifically, if colonization of mine habitats is accompanied by habitat filtering or local adaptation, we would expect mining populations to exhibit genetic differentiation from nearby woodland populations. Alternatively, if persistence is primarily maintained through repeated colonization from surrounding woodlands, we would expect limited genetic divergence but potentially distinct fungal (especially, mycorrhizal) associations that enhance establishment under challenging edaphic conditions. Furthermore, we propose that ongoing seed dispersal contributes to the continued colonization of mine soils, with specific mycorrhizal associates playing an important role in successful establishment and persistence.

To test these hypotheses, we address the following research questions:

1. Do mining individuals of *E. helleborine* associate with distinct mycorrhizal communities compared with adjacent woodland individuals, and are these associations habitat‐specific or shared across sites?
2. Are mining individuals the result of single or multiple colonization events, and is colonization driven by founder effects or ongoing seed dispersal from nearby woods (*i.e*., are mine colonizing individuals closely related or unrelated?) Is genetic diversity reduced in mining individuals relative to woodland individuals, indicating founder effects or a colonization bottleneck during establishment?
3. Do mining individuals in Sardinia and Tuscany represent independent colonization events from local source populations, or could they reflect shared ancestry via dispersal from a previously established mining population?

## MATERIALS AND METHODS

### *Epipactis helleborine* sampling sites

In this study, we investigated population genetic structure and root‐associated fungal communities of *E. helleborine* across two contrasting but spatially adjacent environments: natural woodland sites (F) and anthropogenically disturbed post‐mining sites (M). Sampling was conducted in two regions of Italy, Sardinia (S) and Tuscany (T), representing island and mainland contexts, respectively, thereby capturing both ecological and biogeographic variation (Fig. 1A–C). Within each region, woodland and mining localities occur in close proximity within mixed oak forest landscapes, allowing the effects of local environmental conditions to be examined while minimizing large‐scale climatic variation. All sampling was conducted during the flowering period of *E. helleborine* in May 2023.

The Sardinian mining locality (Fig. S1A) is characterized by pronounced environmental stress, including shallow, poorly developed soils with low organic matter content, strong seasonal aridity, and extreme multimetallic contamination involving Pb, Zn, Cd, As, Tl, and Fe (De Agostini *et al*. 2020). In contrast, the Tuscan mining locality (Fig. S1B) has deeper soils, higher organic content, and more stable moisture availability (E. Sciaudone, pers. obs.). Contamination at the Tuscan site is primarily associated with Pb, Zn, Cu, and Cd, representing a less severe but still substantial anthropogenic stress gradient compared with Sardinia (Dini 2003; Bini & Gaballo 2006).

Adult flowering plants were sampled to ensure accurate species identification (Fig. 1A,B). Within each locality, individuals were collected across the occupied area, with inter‐sample distances generally ranging from tens to hundreds of metres. This fine‐scale sampling design minimizes the influence of broad geographic separation and allows differences in genetic structure and fungal community composition to be attributed primarily to regional and local environmental factors. Root and leaf tissues were collected from the same individuals to enable direct comparisons between genomic and fungal association datasets.

Where accessible, approximately three root apices were collected per individual, although root recovery varied because of rocky substrate conditions. In Sardinia, roots were obtained from 15 woodland and 20 mining individuals, whereas in Tuscany roots were collected from 11 woodland and 18 mining individuals. Leaf material was collected from 96 *E. helleborine* individuals, comprising 24 individuals from each habitat type in both Sardinia and Tuscany.

### Root DNA extraction and fungal amplicon sequencing

Shortly after excavation, root apices were thoroughly rinsed with tap water, treated with a 1% sodium hypochlorite solution following Arnold *et al*. (2003), sonicated to remove surface debris, cut into 2‐cm segments from the original longer pieces, and subsequently frozen. Genomic DNA was then extracted from frozen orchid roots using the NucleoSpin Plant II Mini Kit (Macherey‐Nagel, Düren, Germany), following the manufacturer's protocol. DNA quality and concentration were assessed using a NanoDrop ND‐1000 spectrophotometer (Thermo Scientific, Wilmington, USA).

Fungal identification was based on amplification of the nuclear ribosomal internal transcribed spacer 2 (nrITS2) region using a two‐step PCR protocol. In the first PCR, the fungal ITS2 region was amplified using the primer pair ITS86F (Vancov & Keen 2009) and ITS4 (White *et al*. 1990), optimized for detecting orchid mycorrhizal fungi (OMF). In the second PCR, the same primer pair was used, but each primer carried Illumina overhang adapter sequences at the 5′ end to enable subsequent library preparation and sequencing on the Illumina platform. Each extracted DNA was amplified in triplicate to ensure reproducibility and reduce PCR bias. Amplicons were pooled and sequenced on an Illumina MiSeq platform (Illumina Inc., San Diego, CA, USA) using paired‐end 300 bp reads (PE300).

### Fungal metabarcoding of *E. helleborine* inferred from the amplicon sequencing data

Raw amplicon reads were first assessed for quality using FastQC v0.12.1 (Andrews 2010), and trimmed using Trimmomatic v0.39 (Bolger *et al*. 2014) with a 50 bp sliding window and a minimum average Phred score of 20. Reads shorter than 100 bp post‐trimming were discarded. Overlapping paired‐end reads were merged using PEAR v0.9.6 (Zhang *et al*. 2014), but for taxonomic assignment, only the forward reads were used, due to higher quality scores and broader compatibility with fungal reference databases.

Processed sequences were imported into QIIME2 v2024.10 (Bolyen *et al*. 2019) for downstream analysis. Quality filtering, dereplication, and de novo chimera removal were performed using the VSEARCH plugin (Rognes *et al*. 2016). To validate chimera detection and mitigate potential false‐positives associated with DADA2 (Callahan *et al*. 2016), we conducted a parallel analysis using USEARCH v11 (Edgar 2010). Sequences were clustered into Operational Taxonomic Units (OTUs) at 99% similarity, and taxonomic assignment was performed using a Naive Bayes classifier trained on the UNITE + INSD ITS reference database (version 10.0; Nilsson *et al*. 2019). Ambiguous or low‐confidence annotations were further verified using BLAST+ (Camacho *et al*. 2009) with a conservative e‐value threshold of 1e–5.

To improve taxonomic accuracy and ecological interpretability, we focused on genus‐level diversity, as species‐level assignments were often unreliable and higher level classifications lacked sufficient resolution (Põlme *et al*. 2020). OTUs identified as *E. helleborine*, non‐fungal sequences, or recurrent non‐fungal contaminants were removed prior to analysis. Unidentified OTUs were retained provided they were classified as fungal. All fungal taxa were assigned to ecological functional groups using FungalTraits v1.2 (Põlme *et al*. 2020; Tanunchai *et al*. 2023; Hartvig *et al*. 2024), complemented by manual curation at the genus level. Mycorrhizal fungi included OMF, ectomycorrhizal fungi (ECM), arbuscular mycorrhizal fungi (AMF), and ericoid mycorrhizal fungi (ERM). All remaining taxa, including saprotrophs, endophytes, and putative pathogens, were classified as non‐mycorrhizal fungi (NMY) and unassigned or unidentified as unknown (UNK). This classification was used to generate separate datasets for analyses of total fungal communities and mycorrhizal‐only communities.

After taxonomic filtering, rarefaction analysis was performed to standardize sequencing depth across samples and reduce the influence of rare genera, thereby minimizing sampling bias (Schloss 2024). This was conducted using the *rrarefy* function from the vegan v2.7–2 R package (Oksanen *et al*. 2024), which randomly subsamples each sample to the lowest total read count across the dataset.

Following rarefaction, alpha diversity metrics, including richness, Simpson's index, and Shannon's index, were calculated at the genus level using the *specnumber* and *diversity* functions in vegan. To assess variation in fungal community composition among samples, Bray–Curtis (abundance‐based) and Jaccard (presence–absence‐based) dissimilarity matrices were computed using the *vegdist* function in vegan. Bray–Curtis dissimilarities were calculated from rarefied genus‐level abundance data, whereas Jaccard dissimilarities were calculated from binarized presence–absence matrices to provide a more conservative assessment of community turnover less influenced by variation in sequence abundances.

Ordination and visualization of fungal community patterns were performed using non‐metric multidimensional scaling (NMDS) based on Bray–Curtis dissimilarities, implemented with the *metaMDS* function in the vegan. In addition, principal component analysis (PCA) was conducted on Hellinger‐transformed abundance data using the *decostand* function in vegan followed by *prcomp* in the stats v4.4.2 R package (R Core Team 2024), and principal coordinates analysis (PCoA) was performed using the *cmdscale* function in stats. Differences in fungal community structure across sites (Sardinia *versus* Tuscany) and habitat types (woods *versus* mines) were tested using Permutational Multivariate Analysis of Variance (PERMANOVA) with the *adonis2* function in vegan. When overall PERMANOVA results were significant, pairwise PERMANOVA comparisons among regions and habitat types were conducted using permutation‐based tests, and *P*‐values were adjusted for multiple comparisons using the Holm correction method. Assumptions of homogeneity of group dispersions were verified using *betadisper* followed by *anova.betadisper* functions in vegan to assess significance.

To specifically assess symbiotic fungal associations, the same analytical framework (alpha diversity, dissimilarity metrics, ordination, and PERMANOVA) was applied to the subset of mycorrhizal fungi only (Table S1). This allowed comparison of whether patterns observed in the total fungal community were driven by mycorrhizal fungi.

### Leaf DNA extraction and orchid ddRAD genotyping

Genomic DNA extraction was performed using the cetyl trimethylammonium bromide (CTAB) method on silica‐dried leaf tissues (Doyle & Doyle 1987). DNA quality and quantity were assessed via agarose gel electrophoresis and fluorometry (Qubit 3.0, Thermo Fisher Scientific, Waltham, MA, USA), respectively. DNA samples meeting quality thresholds were normalized to 200 ng in 10 μL of volume. For library preparation, a slight modification to the standard ddRAD protocol (Peterson *et al*. 2012) was used, with the restriction enzymes *Eco*RI and *Taq*I (New England Biolabs, Ipswich, MA, USA). Sequencing was performed on an Illumina NovaSeq 6000 platform (Illumina Inc., San Diego, CA, USA) using paired‐end 150 bp reads (PE150).

### ddRADseq data processing and SNP filtering

Raw paired‐end reads generated from all leaf samples across two independent Illumina NovaSeq runs were demultiplexed and quality filtered using the *process_radtags* module in Stacks v2.66 (Catchen *et al*. 2013), retaining only high‐quality reads. Inline barcodes were used for sample identification, and reads containing ambiguous nucleotides, Phred quality scores below 20, or unreadable or incorrect barcodes were removed. In the absence of a reference genome for *E. helleborine*, RAD loci were assembled de novo using the *denovo_map.pl* pipeline in Stacks. Locus assembly and SNP calling parameters were optimized following Rochette *et al*. (2019) by systematically exploring values of M (within‐individual mismatches) and n (between‐individual mismatches). The parameter combination ustacks –m 1 –M 8 and cstacks –n 2 was selected as it provided the best compromise between read retention, locus completeness, and SNP reliability. All remaining parameters were left at default settings, except for the maximum observed heterozygosity, set to 0.70, and the minimum minor allele frequency (MAF), set to 0.05. To further improve data quality, loci with excessive missing data were filtered using VCFtools v0.1.17 (Danecek *et al*. 2011). SNPs were retained only if present in at least 90% of individuals (‐max‐missing 0.9). Additionally, less stringent thresholds for MAF and missing data were evaluated to assess their effects on SNP recovery.

### Genetic structure of *E. helleborine* inferred from the ddRAD data

To investigate the genetic structure of *E. helleborine*, we employed a combination of multivariate, population genetic, Bayesian clustering, and phylogenetic approaches.

We first conducted a PCA on biallelic SNPs present in at least 25% of individuals using the vcfR v1.15.0 R package (Knaus & Grünwald 2017). To minimize the effects of linkage disequilibrium, we retained only one randomly selected SNP per ddRAD locus. To account for stochasticity in SNP subsampling, we repeated the PCA 50 times, each time selecting a different random SNP per locus. The resulting PC scores were visualized in three dimensions using the scatterplot3d v0.3–44 R package (Ligges & Mächler 2003). To assess broader geographic structuring, an additional PCA was performed including all individuals from Sardinia and Tuscany simultaneously, allowing comparison of the relative contributions of geographic region and habitat type (woodland *versus* mining) to overall genetic variation.

We then estimated population genetic differentiation using Weir and Cockerham's *F*_ST_ via the *pairwise.WCfst* function in the hierfstat v0.5–11 R package (Goudet 2005). This method calculates pairwise *F*_ST_ values among populations, individual inbreeding coefficients, and population‐specific *F*‐statistics (Weir & Goudet 2017).

To quantify the hierarchical distribution of genetic variation, we performed Analysis of Molecular Variance (AMOVA) using the poppr v2.9.8 R package (Kamvar *et al*. 2014). Genetic variance was partitioned among geographic regions (Sardinia *versus* Tuscany), among groups within regions, and within groups based on SNP‐derived genetic distances. In addition, we conducted a PERMANOVA using the vegan package to evaluate the relative effects of geographic region and habitat type (woodland *versus* mining) on overall genetic structure. The analysis was based on Euclidean genetic distances computed from SNP genotypes, and significance was assessed using 9,999 permutations. Interaction effects between region and habitat were also tested.

To further assess population structure and admixture, we applied a Bayesian clustering approach using STRUCTURE v2.3.4 (Pritchard *et al*. 2000). We ran the analysis under an admixture model with correlated allele frequencies, using a burn‐in of 10^4^ iterations followed by 10^5^ MCMC iterations for K values ranging from 2 to 20, with 10 replicates per K. The most likely number of genetic clusters (K) was inferred based on the log‐likelihood and the ΔK method (Evanno *et al*. 2005), using the program STRUCTURE HARVESTER (Earl & vonHoldt 2012). To confirm the clustering results, we also applied the sparse non‐negative matrix factorization (sNMF) method implemented in the LEA v3.16.0 R package (Frichot & François 2015).

Genetic relatedness among individuals was inferred from the SNP dataset using both approximate maximum likelihood (FastTree v2.1.1; Price *et al*. 2010) and maximum likelihood (IQ‐TREE2; Minh *et al*. 2020). The resulting trees were used to visualize genetic distances among individuals, and a pairwise distance heatmap was generated using the pheatmap v1.0.13 R package (Kolde 2019). Habitat type (woodland *versus* mining) and geographic origin (Sardinia *versus* Tuscany) were mapped onto the phylogenies to assess the presence of clustering by environment or location.

Finally, Mantel tests were performed with the *mantel* function in vegan to evaluate whether genetic relatedness among *E. helleborine* individuals (based on SNP‐derived genetic distance matrices) correlated with fungal community dissimilarity (Bray–Curtis and Jaccard), thereby testing the relationship between the level of relatedness of host genotypes and their mycorrhizal composition (Jacquemyn *et al*. 2016; Suetsugu *et al*. 2017).

## RESULTS

### Fungal metabarcoding of *E. helleborine* inferred from the amplicon sequencing data

**Fig. 2:**
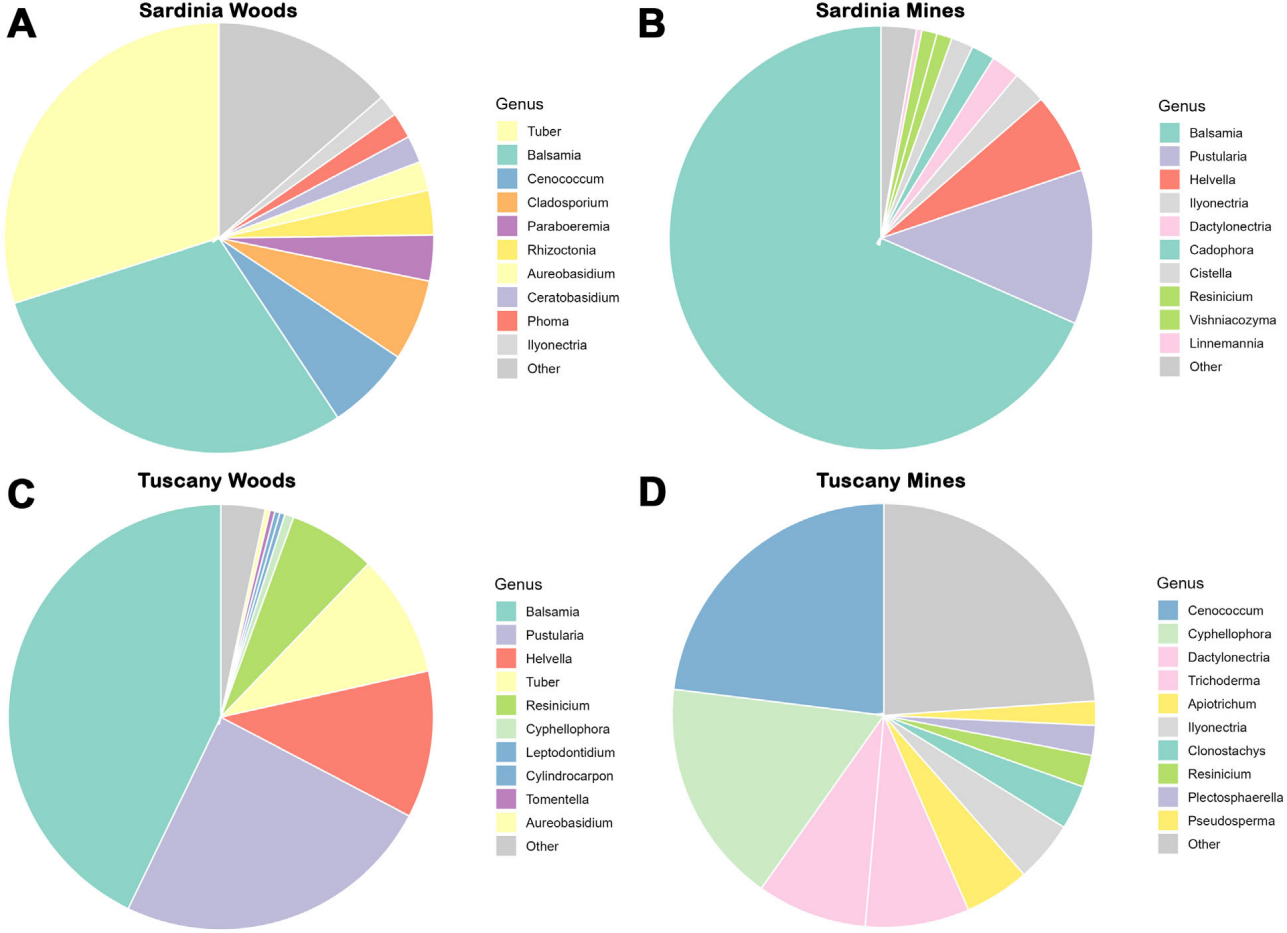
Fungal community composition in *Epipactis helleborine* roots across habitats and regions. Pie charts show the relative abundance of dominant fungal genera associated with individual root samples from mining and woodland sites in Sardinia and Tuscany. (A) Sardinia woods, (B) Sardinia mine, (C) Tuscany woods, and (D) Tuscany mine.

We analysed fungal ITS2 barcoding data from 42 *Epipactis helleborine* individuals sampled across mining and woodland habitats in Sardinia and Tuscany; after quality filtering (BLAST identity >99%, e‐value <1e–5, alignment length >100 bp), 37 samples (Sardinia woods = 12, Sardinia mines = 12; Tuscany woods = 6, Tuscany mines = 7) yielded sufficient sequence data for downstream analyses (Table S1). Both USEARCH and VSEARCH pipelines recovered highly consistent OTU sets, supporting the robustness of feature inference (Table S1). Genus‐level abundances per sample ranged from 1 to 117, with sequencing depths spanning approximately 900–90,000 reads, and more than 300 fungal genera represented by fewer than 10 reads, indicating a pronounced long tail of rare taxa (Table S1). *Balsamia* (specifically, *B. vulgaris*) was consistently detected across most samples, with read counts ranging from 11 to 75,475 (Fig. 2A–D; Table S1). Fungal community composition varied among habitats and regions (Fig. 2A–D; Table S1).

**Fig. 3:**
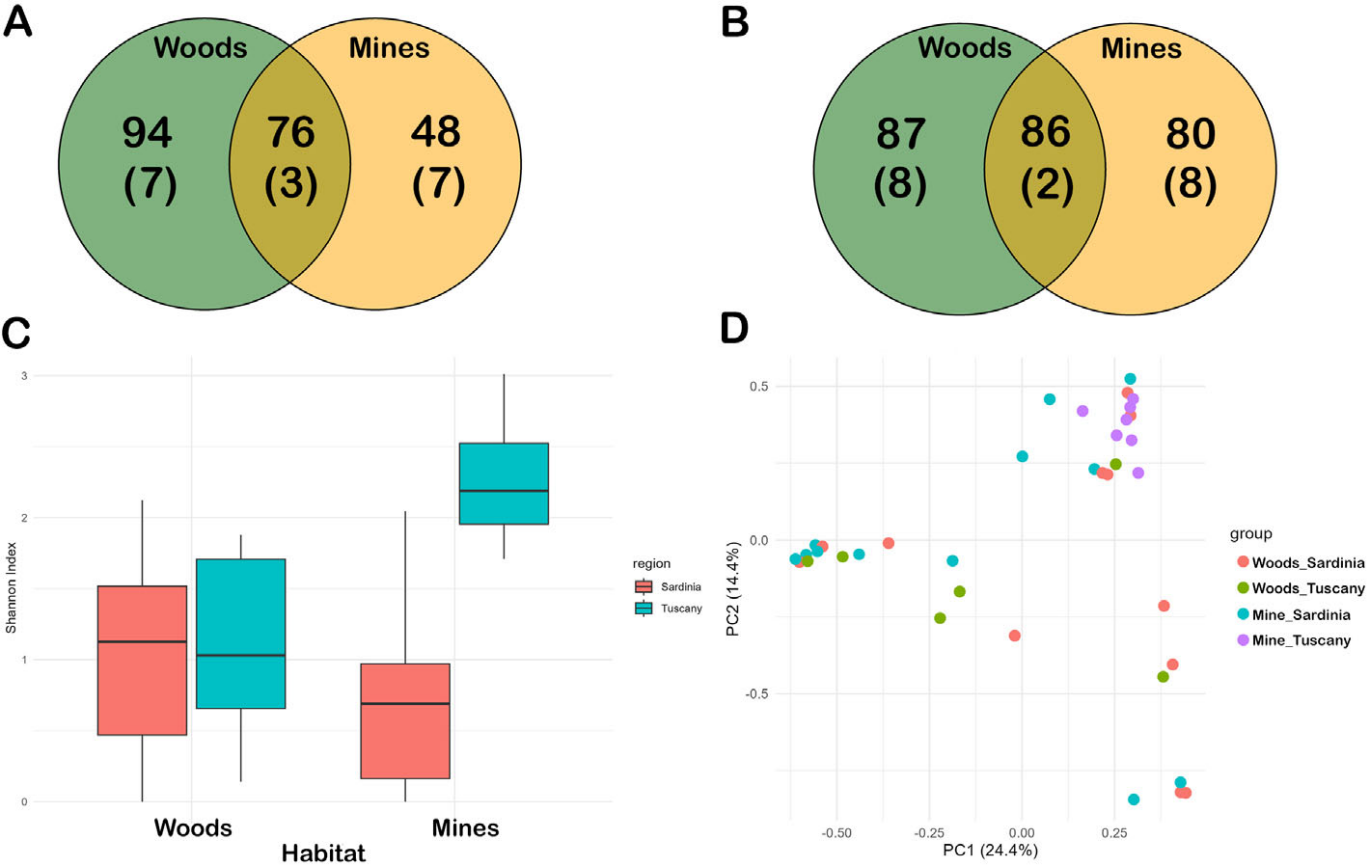
Fungal colonization patterns of *Epipactis helleborine* in Sardinia and Tuscany. (A) Venn diagram showing the total number of fungal genera associated with *E. helleborine* in mining and woodland sites in Sardinia, along with the 10 most abundant genera unique to each habitat and those shared between them (in parentheses); (B) Venn diagram displaying the same information for Tuscany; (C) Shannon diversity index of fungal communities in woodland and mining sites across both regions; and (D) Principal component analysis (PCA) ordination of fungal community composition, highlighting habitat‐based clustering across Sardinia and Tuscany.

After rarefaction to standardize sampling effort, alpha diversity metrics (genus richness, Shannon, and Simpson indices) revealed no significant richness differences between mine and woodland habitats in Sardinia, whereas Tuscany mine site consistently exhibited higher alpha diversity than Tuscany woodland site across all indices. Habitat‐specific analyses identified 94 fungal genera unique to Sardinian woods, 48 unique to Sardinian mines, and 76 shared between habitats, whereas Tuscany contained 87 genera unique to woods, 80 unique to mines, and 86 shared between habitats (Fig. 3A,B). Per‐sample genus richness was highest in Tuscany mines (median = 16), intermediate in Sardinian woods and mines (both median = 6), and lowest in Tuscany woods (median = 3). In contrast, Tuscany woods harboured the highest cumulative richness across all samples (173 genera), indicating elevated beta diversity and substantial compositional turnover. Similar patterns were observed for Shannon and Simpson diversity indices, with Tuscany mines showing the highest diversity (median Shannon ≈ 2.1; Simpson ≈ 0.77), Sardinian woods and mines displaying intermediate diversity (Shannon ≈ 1.2 and 1.1; Simpson ≈ 0.62 and 0.60), and Tuscany woods exhibiting the lowest diversity (Shannon ≈ 0.49; Simpson ≈ 0.24; Fig. 3C).

When analyses were restricted to functional groups, mycorrhizal fungi (including ectomycorrhizal fungi, ECM; arbuscular mycorrhizal fungi, AMF; ericoid mycorrhizal fungi, ERM; and orchid mycorrhizal fungi, OMF) exhibited lower alpha diversity compared with the whole fungal community. The genus richness was highest in Tuscany sites, with almost similar values observed in woodland and mine habitats (Tuscany woods = 7.17; Tuscany mines = 7.00). In contrast, Sardinian sites exhibited lower taxonomic richness overall, particularly in mine habitats (Sardinia woods = 5.36; Sardinia mines = 3.23). A similar pattern was observed for Shannon diversity, with Tuscany mine communities showing the highest diversity (1.17), followed by Tuscany woods (0.82), while Sardinian woods (0.41) and especially Sardinian mines (0.24) exhibited substantially lower diversity. This suggests reduced species evenness and/or dominance effects in Sardinian fungal assemblages, particularly in mining habitats. Simpson diversity showed consistent trends, with highest values in Tuscany mines (0.55) and Tuscany woods (0.45), and markedly lower values in Sardinian woods (0.22) and Sardinian mines (0.14). Together, these patterns indicate that Tuscany habitats support more diverse and more even mycorrhizal communities, whereas Sardinian communities, especially in mine environments, are characterized by lower diversity and stronger dominance structure.

Ordination analyses based on Bray–Curtis dissimilarities revealed limited habitat‐ or site‐specific structuring of fungal community composition, although samples from Tuscan mines formed a loosely defined cluster in the PCA plot (Fig. 3D). PERMANOVA analyses showed that the interaction between habitat and geographic location significantly explained variation in fungal community composition (R^2^ = 0.077, F = 3.06, *P* = 0.003), whereas habitat alone (*P* = 0.110) and region alone (*P* = 0.145) were not significant. These findings indicate that the influence of habitat on fungal assemblages differed between Sardinia and Tuscany rather than representing a uniform habitat effect across regions. Re‐analysis using the more conservative Jaccard dissimilarity metric based on presence/absence data recovered broadly similar overall patterns (overall PERMANOVA: R^2^ = 0.145, F = 1.93, *P* = 0.003), although several pairwise habitat contrasts became weaker and were no longer significant after multiple testing correction. Beta‐dispersion analyses detected no significant heterogeneity of variances among groups (Bray–Curtis: F = 0.15, *P* = 0.70), indicating that the observed PERMANOVA patterns were not driven by unequal within‐group dispersion. Pairwise PERMANOVA analyses based on Bray–Curtis dissimilarities further revealed context‐dependent differentiation among fungal communities. The strongest divergence was observed between mining habitats across regions, with Sardinia mines differing significantly from Tuscany mines (R^2^ = 0.144, F = 3.04, *P* = 0.003, *P*‐adj = 0.018), a pattern that was also recovered using Jaccard dissimilarities (R^2^ = 0.111, F = 2.25, *P* = 0.005, *P*‐adj = 0.030). In addition, Bray–Curtis analyses detected a relatively strong within‐region habitat effect in Tuscany, where woodland and mining communities differed significantly (R^2^ = 0.197, F = 2.71, *P* = 0.003, *P*‐adj = 0.018). However, this contrast became weaker under Jaccard dissimilarity and was no longer significant after multiple testing correction (*P*‐adj = 0.174), suggesting that the observed differentiation is influenced primarily by shifts in relative abundances rather than complete replacement of fungal taxa. At the uncorrected level, Sardinia woods and Sardinia mines also differed significantly under both Bray–Curtis (F = 1.85, *P* = 0.043) and Jaccard (F = 1.66, *P* = 0.025) analyses, although neither comparison remained significant after correction for multiple testing. Comparisons between Sardinia woods and Tuscany mines showed moderate differentiation, whereas woodland communities were comparatively conserved across regions, with no significant difference detected between Tuscany woods and Sardinia woods under either dissimilarity metric.

Community composition of mycorrhizal fungi differed significantly among the four habitat–region groups (Tuscany woods, Tuscany mines, Sardinia woods, and Sardinia mines) as indicated by Bray–Curtis‐based PERMANOVA (R^2^ = 0.158, F = 2.06, *P* = 0.003). Tests for homogeneity of multivariate dispersion were not significant (Bray–Curtis: F = 0.48, *P* = 0.687), confirming that these differences reflected shifts in community composition rather than unequal within‐group variability. Pairwise Bray–Curtis comparisons revealed significant differentiation between Tuscany woodland and Tuscany mine communities (F = 2.85, R^2^ = 0.206, *P*‐adj = 0.006) and between Sardinian mine and Tuscany mine communities (F = 3.41, R^2^ = 0.159, *P*‐adj = 0.015), whereas other comparisons were either non‐significant or only marginally significant after correction for multiple testing. Analyses based on Jaccard distances, which emphasize taxon turnover, revealed significant differences between Sardinian mine and Sardinian woodland communities (F = 2.69, R^2^ = 0.109, *P*‐adj = 0.030), between Sardinian mine and Tuscany mine communities (F = 3.14, R^2^ = 0.148, *P*‐adj = 0.024), and between Sardinian woodland and Tuscany woodland communities (F = 2.21, R^2^ = 0.129, *P*‐adj = 0.030). Together, these results demonstrate significant differences in mycorrhizal community composition among habitat–region groups, although the specific patterns detected varied between abundance‐based (Bray–Curtis) and presence–absence (Jaccard) analyses.

### ddRAD sequence reads and coverage

Leaf samples yielded 1,605,700,138 raw paired‐end reads across two independent Illumina NovaSeq runs; following demultiplexing and quality filtering in Stacks, 789,586,368 reads were retained for downstream analyses.

For the Sardinia site, a total of 556,064 loci were successfully assembled into contigs, with an average contig length of 296.5 bp. All these loci were subsequently genotyped, with an average effective per‐sample coverage of 20.8× (±5.2× SD; range: 11.4 × −48.9×). Across these loci, 134,049 variants were identified, of which 3,141 were retained after filtering. After removing loci with missing data, 378 SNP loci remained for downstream analyses.

For the Tuscany site, a total of 271,181 loci were successfully assembled into contigs, with an average contig length of 283.47 bp. All these loci were subsequently genotyped, with an average effective per‐sample coverage of 18.5× (±4.7× SD; range: 7.36 × −45.4×). Across these loci, 72,018 variants were identified, of which 2,037 were retained after filtering. After removing loci with missing data, 223 SNP loci remained for downstream analyses.

Overall, after quality filtering of the ddRAD SNP dataset, the following samples were retained for downstream analyses: Sardinia woods (*n* = 20), Sardinia mines (*n* = 21), Tuscany woods (*n* = 21), and Tuscany mines (*n* = 22).

### Genetic structure of *E. helleborine* inferred from the ddRAD SNP data

**Fig. 4:**
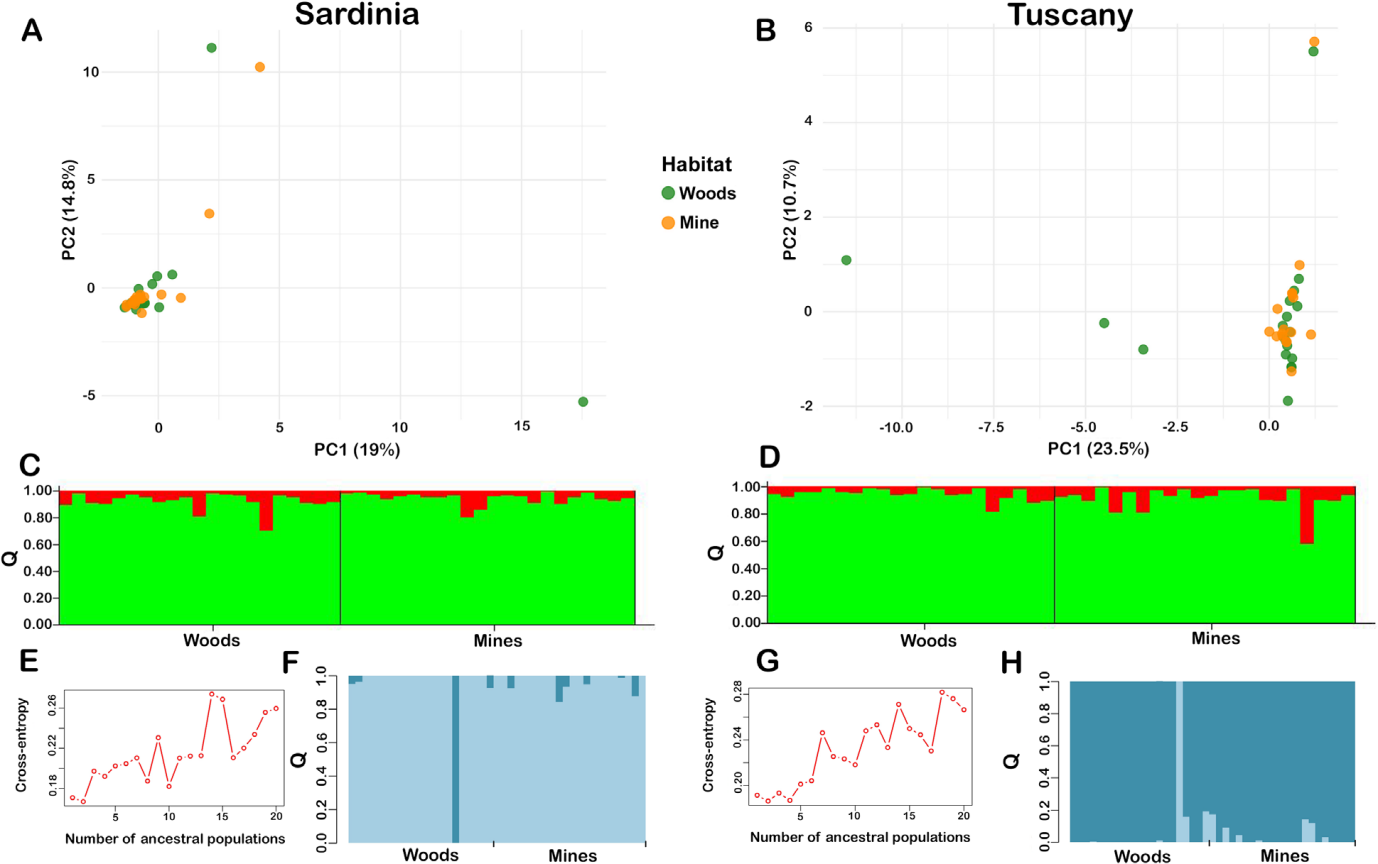
Genetic structure of *Epipactis helleborine* in Sardinia and Tuscany. (A, B) Principal component analysis (PCA) of genetic variation among individuals from (A) Sardinia and (B) Tuscany, (C, D) STRUCTURE plots showing individual ancestry proportions for (C) Sardinia and (D) Tuscany, (E–G) Cross‐entropy plots used to determine the optimal number of ancestral populations in (E) Sardinia and (G) Tuscany, and (F, H) Barplots of individual ancestry coefficients for the best‐supported number of clusters in (F) Sardinia and (H) Tuscany. The Y‐axis in panels C, D, F, and H represents the estimated ancestry proportion (membership coefficient, Q).

The PCA of biallelic SNPs revealed no clear genetic clustering between individuals from mining and woods habitats in either Sardinia or Tuscany (Fig. 4A,B). For Sardinia, the first principal component (PC1) accounted for 19.0% of the genetic variation, while PC2 explained 14.8% (Fig. 4A). For Tuscany, the first principal component (PC1) accounted for 23.5% of the genetic variation, while PC2 explained 10.7% (Fig. 4B). Replicated PCA analyses using different random SNPs per locus consistently supported the absence of strong population‐level structure. When all samples from Sardinia and Tuscany were analysed together, PCA revealed clear geographic separation between the two regions, with PC1 (11.45%) primarily distinguishing Sardinian and Tuscan populations.

For Sardinia, the genetic statistics indicate very low and slightly negative population differentiation between the mine and woods groups, with a pairwise *F*_ST_ of 0.005, suggesting virtually no genetic structure. Observed heterozygosity (H_o_ = 0.6051) is considerably higher than the expected heterozygosity within groups (H_e_ = 0.3914), indicating a strong excess of heterozygotes. Total genetic diversity across groups (H_t_ = 0.3911) closely matches the within‐group diversity, reflected in a slightly negative D_st_ (−0.0003) and an overall *F*_ST_ near zero (−0.0007), further supporting the lack of genetic differentiation. The strongly negative inbreeding coefficient (*F*_IS_ = −0.5460) confirms heterozygote excess within groups.

For Tuscany site, the population genetic summary indicates a low but slightly positive genetic differentiation between the mine and woods groups, with a pairwise *F*_ST_ of 0.0122. Observed heterozygosity (H_o_ = 0.6092) remains substantially higher than the expected heterozygosity within groups (H_e_ = 0.3938), suggesting an excess of heterozygotes overall. The total genetic diversity across groups (H_t_ = 0.3949) is very close to within‐group diversity, reflected in a small positive D_st_ (0.0011) and a low overall *F*_ST_ (0.0027), indicating minimal population structure. The strongly negative inbreeding coefficient (*F*_IS_ = −0.5470) is in agreement with heterozygote excess within groups.

Hierarchical AMOVA revealed significant genetic structuring across the study area. Most genetic variation was distributed within groups (82.7%), whereas 16.9% of the total variation was attributable to differences between Sardinian and Tuscan samples. In contrast, variation among groups within regions accounted for only 0.3% of the total variance (Table S2). Corresponding Phi‐statistics indicated moderate genetic differentiation between regions (Φ_RT_ = 0.169), while differentiation among groups within regions was negligible (Φ_PR_ = 0.004). PERMANOVA analyses produced consistent results (Tables S2–S4). Geographic region explained 10.9% of the total genetic variation and significantly influenced population structure (R^2^ = 0.109, F = 8.17, *P* < 0.001). In contrast, habitat type accounted for only 1.5% of the variation and was not significant (R^2^ = 0.015, F = 1.10, *P* = 0.257). Likewise, the interaction between region and habitat explained only 1.3% of the variation and was not significant (R^2^ = 0.013, F = 1.01, *P* = 0.378).

Bayesian clustering analysis using STRUCTURE also failed to detect distinct substructure between mining and woods groups. The most likely number of genetic clusters was inferred to be K = 2 in both Sardinia and Tuscany, but individuals showed mixed ancestry regardless of habitat type (Fig. 4C,D). Although the ΔK method supported K = 2, this result likely reflects MAF variation or methodological bias, as the ΔK method cannot evaluate K = 1 and often favours K = 2 even in panmictic populations (Fig. 4E,G). Visual inspection of the barplots further supported the absence of meaningful structure, with individuals assigned to both clusters in roughly equal proportions. These results were corroborated by sNMF clustering, which likewise indicated no strong genetic partitioning between groups from different habitats (Fig. 4F,H).

**Fig. 5:**
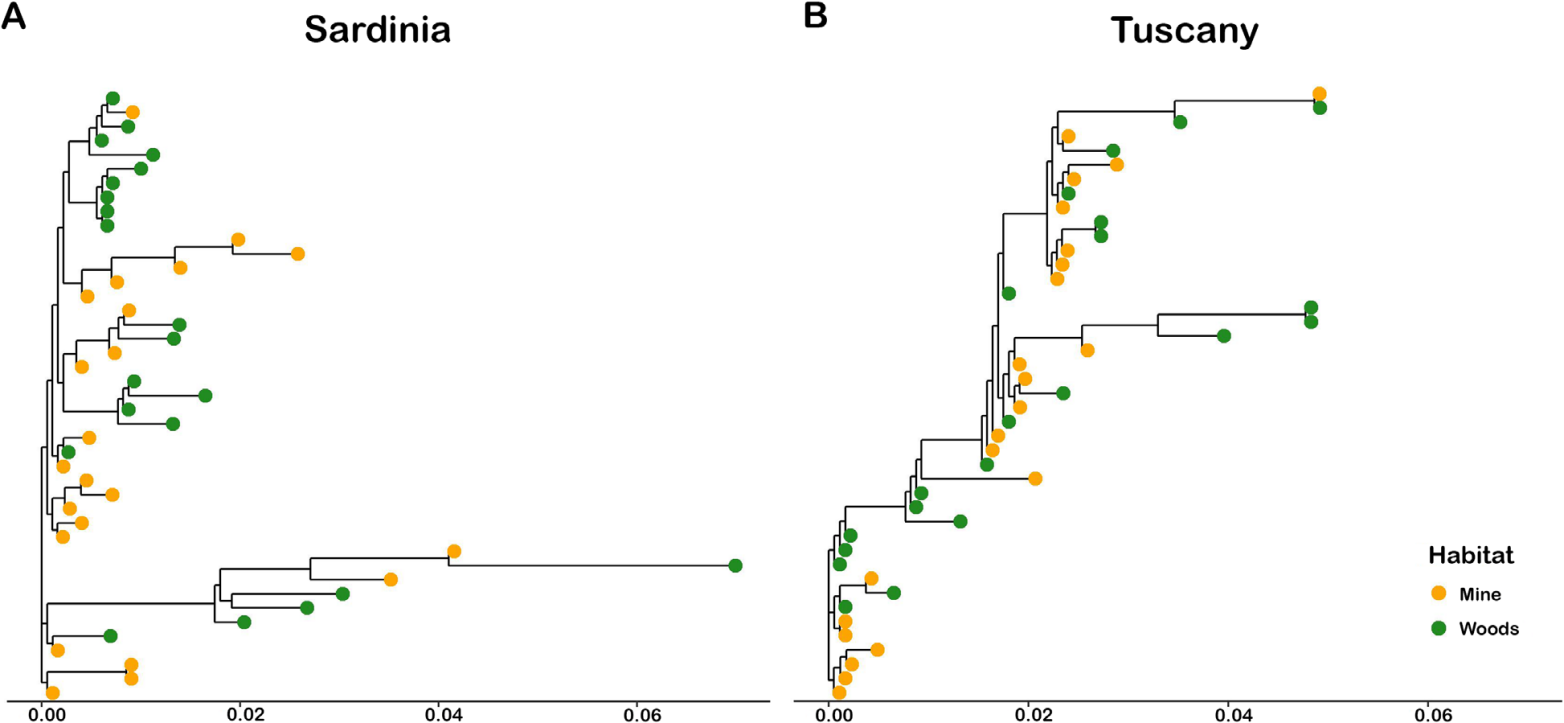
Genealogical analyses of *Epipactis helleborine* individuals from Sardinia and Tuscany. Approximate maximum likelihood trees based on SNP data for individuals from Sardinia (A) and Tuscany (B). In both trees, the X‐axis represents cumulative branch length (genetic divergence) from the root to the tips.

Maximum likelihood phylogenetic trees generated using both Fasttree and IQ‐TREE2 showed no clustering of individuals by habitat type (mine versus woods) in either site (Fig. 5A,B). Individuals from both habitat types were interspersed throughout the tree, indicating a lack of phylogenetic separation between mining and woods groups (Fig. 5A,B). Heatmaps of pairwise genetic distances further supported this pattern, revealing no consistent clustering by habitat (Fig. S2A,B).

Finally, Mantel tests comparing fungal community dissimilarity matrices with host genetic distances showed no significant correlation using either Bray–Curtis (Mantel r = 0.082, *P* = 0.155) or Jaccard (Mantel r = 0.020, *P* = 0.391) distances, indicating that fungal community composition is not structured by host genetic relatedness. Further, Mantel tests comparing mycorrhizal community dissimilarity matrices with host genetic distances also showed no significant correlation using either Bray–Curtis (Mantel r = 0.088, *P* = 0.144) or Jaccard (Mantel r = 0.026, *P* = 0.35) distances.

## DISCUSSION

### Fungal associates and functional adaptability of *E. helleborine*

Compared with the relatively weak genetic structuring detected among individuals, root‐associated total fungal and mycorrhizal communities exhibited habitat‐associated differences that were moderate and region‐dependent (Figs. 2 and 3). In both regions, mining and woodland habitats shared only a subset of fungal genera, with partial overlap among dominant taxa (Figs. 2 and 3A,B). These patterns indicate compositional turnover between habitats, particularly in Tuscany, and suggest that post‐mining environments support fungal assemblages that are distinct, but not completely separated, from those of adjacent woodland communities.

*Epipactis helleborine* exhibited considerable symbiotic flexibility, associating with a diverse range of fungi, including taxa not previously reported from this orchid. This observation is consistent with earlier studies demonstrating broad fungal associations and trophic flexibility in *E. helleborine* (Ogura‐Tsujita & Yukawa 2008; Jacquemyn *et al*. 2016; Valencia *et al*. 2021). Among the dominant fungal (ectomycorrhizal) associates, *Tuber* occurred in both Sardinian and Tuscan populations, although relative abundances varied among habitats and regions. We additionally detected *Balsamia* in several individuals, representing a novel record of this genus in association with *E. helleborine*. All occurrences were associated with oak‐dominated woodland habitats or their remnants, consistent with the known ecology of *Balsamia* species (Chavdarova *et al*. 2011). Although *Balsamia* has not traditionally been regarded as a common orchid mycorrhizal lineage, members of the genus have previously been recovered from germinating orchid seeds, mycorrhizal seedlings, and orchid‐associated ectomycorrhizal communities of *Epipactis* and *Cephalanthera* (Bidartondo & Read 2008; Southworth *et al*. 2018). These findings suggest that *Balsamia* may participate in orchid–fungus interactions under natural conditions. However, because our conclusions are based on ITS metabarcoding, we cannot determine whether *Balsamia* (specifically, *B. vulgaris*) functions as a true mycorrhizal symbiont, an endophyte, a secondary colonizer, or another root‐associated fungus (Selosse *et al*. 2022). Consequently, we interpret *B. vulgaris* as a putative orchid‐associated fungus rather than a confirmed mycorrhizal partner.

Woodland individuals in both Sardinia and Tuscany were predominantly associated with ectomycorrhizal genera such as *Balsamia*, *Helvella*, and *Tuber*, reflecting relatively intact forest symbiotic networks. In contrast, mining habitats, particularly in Tuscany, were characterized by increased abundances of ectomycorrhizal genera such as *Cadophora*, *Cenococcum*, and *Pustularia* along with stress‐tolerant or opportunistic NMY, including *Dactylonectria*, *Ilyonectria*, *Plectosphaerella*, and *Trichoderma*, taxa frequently associated with disturbed soils (Fig. 2; Table S1; Manici *et al*. 2018). Sardinian mine site retained a greater representation of ectomycorrhizal fungi, suggesting regional differences in fungal community resilience under edaphic stress (Table S1). Notably, classical orchid‐associated fungi such as *Rhizoctonia* and *Ceratobasidium* were detected only in woodland populations from Sardinia and were absent or rare in mining habitats (Table S1). Because our sampling focused exclusively on adult plants, we cannot exclude the possibility that these fungi are more important during germination and early developmental stages.

The observed differences between mining and woodland communities support the view that local environmental conditions influence the fungal partners available to orchids. Fungal community composition varied according to habitat type, whereas woodland communities showed comparatively consistent composition across regions. Despite regional variation, habitat‐specific fungal assemblages occurred repeatedly in both Sardinia and Tuscany, suggesting convergent ecological filtering. These results indicate that *E. helleborine* is capable of associating with locally available fungal partners rather than relying on a narrowly defined set of symbionts. Overall, mining and woodland individuals show distinct but overlapping mycorrhizal communities, indicating habitat‐driven filtering rather than complete turnover. Such flexibility may contribute to the successful establishment of this orchid in degraded and metal‐contaminated environments. This interpretation is consistent with previous studies documenting ecological shifts in orchid–fungal associations following habitat disturbance (Tešitelová *et al*. 2012; Koob 2021; Valencia *et al*. 2021) and with evidence that Sardinian populations tolerate elevated heavy metal concentrations despite measurable physiological costs (De Agostini *et al*. 2020).

### Genetic structure of individuals from mining and woodland habitats

Despite notable ecological differences between mining and woodland habitats, individuals of *E. helleborine* showed little genetic differentiation. Genealogical analyses, PCA, AMOVA, STRUCTURE, and sNMF consistently revealed extensive overlap between habitats, with individuals clustering primarily according to geographic region rather than habitat type (Figs. 4 and 5; Figs. S2 and S3). Comparable levels of heterozygosity and allelic richness further argue against strong founder effects or severe bottlenecks during colonization. Together, these results support repeated colonization of mining habitats from nearby woodland populations under ongoing gene flow.

The weak genetic structuring observed in *E. helleborine* is consistent with its reproductive biology. The species produces numerous wind‐dispersed seeds capable of long‐distance dispersal and is pollinated by a broad range of insects that readily move between natural and disturbed habitats (Rewicz *et al*. 2017). These traits likely promote high connectivity among populations and facilitate repeated establishment in newly available habitats. Similar patterns of extensive admixture and limited genetic differentiation have been reported in other orchids and edaphically contrasting systems, where ecological divergence can occur despite ongoing gene flow (O'Dell & Rajakaruna 2011; Phillips *et al*. 2012; Ahatović Hajro *et al*. 2024).

The absence of strong habitat‐associated genetic structure contrasts with the marked differences observed in fungal communities (Tables S2–S4). This decoupling suggests that habitat colonization in *E. helleborine* is not constrained primarily by host genetic differentiation but instead may depend on the ability of individuals to establish associations with locally available fungal partners. Such a strategy is likely facilitated by the species' broad ecological amplitude, generalized pollination system, and symbiotic flexibility, characteristics that distinguish it from more specialized orchid taxa (Jacquemyn *et al*. 2015). Together, these patterns reject a scenario of single colonization events followed by strong founder effects or bottlenecks in mining habitats, instead supporting repeated establishment from nearby woodland populations under ongoing gene flow. The strong regional genetic structure further indicates that mining populations in Sardinia and Tuscany originated independently from their respective local woodland populations, rather than from dispersal among previously established mining populations (Fig. S3).

### From disturbance to resilience: Ecological implications

Collectively, our results indicate that successful colonization of post‐mining habitats by *E. helleborine* is associated with ecological flexibility in mycorrhizal associations rather than genetic differentiation from nearby woodland populations. Despite substantial differences in mycorrhizal community composition between mining and woodland habitats, genetic diversity remained comparable, indicating that repeated seed dispersal and establishment, rather than long‐term isolation, underpin population persistence in mining environments. These findings reinforce the growing body of evidence that some orchids possess considerable ecological and symbiotic flexibility (Jacquemyn *et al*. 2016; Li *et al*. 2021). In *E. helleborine*, the capacity to associate with diverse mycorrhizal partners appears to facilitate establishment in environments characterized by altered soils, elevated metal concentrations, and disturbed microbial communities. Such flexibility may allow populations to persist where more specialized orchids would be unable to establish. The mining landscapes of Sardinia and Tuscany therefore provide a useful model for understanding plant adaptation and persistence in human‐altered environments. Future studies should focus on determining the functional significance of dominant fungal associates, particularly taxa such as *Balsamia*, through microscopy, stable‐isotope approaches, metatranscriptomics, and symbiotic germination experiments. Integrating these approaches with broader geographic sampling will provide a more comprehensive understanding of orchid–fungus interactions and their role in facilitating plant resilience under environmental change.

## AUTHOR CONTRIBUTIONS

AA contributed to data analysis and writing; ES was involved in sampling and genetic analyses; AB and PC contributed to conceptualization and sampling; DC performed molecular analyses; SC was responsible for conceptualization, writing, and funding acquisition.

## CONFLICTS OF INTEREST

The authors affirm that there are no conflicts of interest relevant to this work.

## FUNDING INFORMATION

This work was supported by ‘National Recovery and Resilience Plan (NRRP)’, Mission 4 Component 2 Investment 1.4—Call for tender No. 3138 of 16 December 2021, rectified by Decree n.3175 of 18 December 2021 of the Ministero dell'Università e della Ricerca funded by the European Union—NextGenerationEU; Award Number: Project code CN_00000033, Concession Decree No. 1034 of 17 June 2022 adopted by the Italian Ministry of University and Research, CUP H43C22000530001, Project title ‘National Biodiversity Future Center—NBFC’. Donata Cafasso was funded under the Project FRA‐HYSENSING CUP E65F22000050001, financially supported by the University of Naples Federico II.

## Supporting information


**Fig. S1.** Woodland and post‐mining sampling locations and sampled individuals of *Epipactis helleborine* included in this study. (A) Leaf and root sampling sites in Sardinia (woods: 39.37513–39.37515° N, 8.59246° E; mines: 39.36790–39.36821° N, 8.60753–8.60800° E) and (B) Tuscany (woods: 43.07399–43.07714° N, 10.61200–10.61804° E; mines: 43.07464–43.07624° N, 10.61368–10.61540° E). Scale bars represent 150 metres in both panels.

**Fig. S2.** Genetic distance analyses of *Epipactis helleborine* individuals across Sardinia and Tuscany. Heatmap of pairwise genetic distances among individuals from Sardinia (A) and Tuscany (B). In the sample names, ‘F’ denotes woods and ‘M’ denotes mines.

**Fig. S3.** Combined maximum likelihood phylogenetic tree and genetic distance heatmap of *Epipactis helleborine* leaf samples from Sardinia and Tuscany. The tree illustrates the genetic relationships among individuals based on ddRAD‐seq data, with branch tips coloured by habitat (woods *versus* mines) and region. The adjacent heatmap shows pairwise genetic distances, highlighting patterns of similarity and divergence across habitats and sites. In the sample names, ‘SAR’ denotes Sardinia and ‘TUS’ denotes Tuscany, while ‘F’ refers to woods and ‘M’ to mines.

**Table S1.** Taxonomic composition and relative abundance of fungal genera detected in *Epipactis helleborine* roots by fungal amplicon sequencing across woodland and abandoned mining habitats in Sardinia and Tuscany. Functional assignments are based on trophic mode classifications: AMF = arbuscular mycorrhizal fungi, ECM = ectomycorrhizal fungi, ERM = ericoid mycorrhizal fungi, NMY = non‐mycorrhizal fungi, OMF = orchid mycorrhizal fungi, and UNK = unassigned/unknown functional group.

**Table S2.** Hierarchical AMOVA of *Epipactis helleborine* SNP data.
**Table S3.** Associated Phi‐statistics in the AMOVA analysis.
**Table S4.** PERMANOVA testing effects of region and habitat on orchid genetic structure.

## Data Availability

The data supporting the findings of this study are available from the corresponding author upon request.
