# Supplementary material for: Underground allies: Mycorrhizal flexibility facilitates orchid occurrence in post‐mining landscapes
Source: Plant Biol (Stuttg). 2026 Jul 27;28(6):1899–911. doi: 10.1111/plb.70269 (PMC13586638; doi:10.1111/plb.70269)
Supplement: Supplementary file 1 [file PLB-28-1899-s004.docx]

**Table S2. Hierarchical AMOVA of *Epipactis helleborine* SNP data.**

| **Source of Variation** | **df** | **Variance Component (σ²)** | **Percentage of Variation (%)** |
| --- | --- | --- | --- |
| Among regions (Sardinia vs Tuscany) | 1 | 1.314 | 16.94 |
| Among populations within regions | 2 | 0.027 | 0.34 |
| Within populations | 65 | 6.420 | 82.72 |
| **Total** | **68** | **7.761** | **100.00** |

**Table S3. Associated Phi-statistics in the AMOVA analysis.**

| **Statistic** | **Value** |
| --- | --- |
| Φ_RT_ (among regions) | 0.169 |
| Φ_PR_ (among populations within regions) | 0.004 |
| Φ_PT_ (overall genetic structure) | 0.173 |

**Table S4. PERMANOVA testing effects of region and habitat on orchid genetic structure.**

| **Source** | **df** | **Sum of Squares** | **R²** | **F** | ***P*-value** |
| --- | --- | --- | --- | --- | --- |
| Region | 1 | 168.65 | 0.109 | 8.172 | <0.001 |
| Habitat | 1 | 22.68 | 0.015 | 1.099 | 0.257 |
| Region × Habitat | 1 | 20.93 | 0.013 | 1.014 | 0.378 |
| Residual | 65 | 1341.52 | 0.863 | – | – |
| **Total** | **68** | **1553.78** | **1.000** | – | – |
